# Sevoflurane ameliorates intestinal ischemia-reperfusion-induced lung injury by inhibiting the synergistic action between mast cell activation and oxidative stress

**DOI:** 10.3892/mmr.2015.3527

**Published:** 2015-03-23

**Authors:** CHENFANG LUO, DONGDONG YUAN, WEICHENG ZHAO, HUIXIN CHEN, GANGJIAN LUO, GUANGJIE SU, ZIQING HEI

**Affiliations:** 1 Department of Anesthesiology, The Third Affiliated Hospital, Sun Yat-Sen University, Guangzhou, Guangdong 510630, P.R. China; 2 Department of Anesthesiology, The First People’s Hospital of Foshan, Foshan, Guangdong 528000, P.R. China

**Keywords:** mast cell, sevoflurane, acute lung injury, intestinal ischemia-reperfusion, oxidative stress, NADPH enzyme

## Abstract

Preconditioning with sevoflurane (SEV) can protect against ischemia-reperfusion injury in several organs, however, the benefits of SEV against acute lung injury (ALI), induced by intestinal ischemia-reperfusion (IIR), and the underlying mechanisms remain to be elucidated. The present study was designed to investigate the effects of SEV preconditioning on IIR-mediated ALI and the associated mechanisms in a rat model. Female Sprague-Dawley rats treated with 2.3% SEV or apocynin (AP), an inhibitor of NADPH oxidase, were subjected to 75 min superior mesenteric artery occlusion followed by 2 h reperfusion in the presence or absence of the mast cell degranulator compound 48/80 (CP). SEV and AP were observed to downregulate the protein expression levels of p47^phox^ and gp91^phox^ in the lungs of normal rats. IIR resulted in severe lung injury, characterized by significant increases in pathological injury scores, lung wet/dry weight ratio, protein expression levels of p47^phox^, gp91^phox^ and ICAM-1, the presence of hydrogen peroxide, malondydehyde and interleukin-6, and the activity of myeloperoxidase. In addition, significant reductions were observed in the expression of prosurfactant protein C, accompanied by an increase in MC degranulation, demonstrated by significant elevations in the number of mast cells, expression levels of tryptase and the concentration of β-hexosaminidase. These changes were further augmented in the presence of CP. In addition, SEV and AP preconditioning significantly alleviated the above alterations induced by IIR alone or in combination with CP. These findings suggested that SEV and AP attenuated IIR-induced ALI by inhibiting NADPH oxidase and the synergistic action between oxidative stress and mast cell activation.

## Introduction

Intestinal ischemia-reperfusion (IIR) is a challenging clinical syndrome occurring in patients undergoing major surgery, including cardiac surgery and liver transplantation (1–4). IIR leads to local injury, but also induces severe remote organ injury, particularly acute lung injury (ALI), which is associated with high rates of mortality (5).

Previous studies have demonstrated that oxidative stress is important in IIR-mediated ALI (6,7). Reactive oxygen species (ROS), predominantly from neutrophil sequestration, contribute to ALI (8) and high concentrations of ROS in the bronchoalveolar lavage fluid are correlated with the severity of ALI in rabbits induced by hemorrhagic shock and resuscitation (9). The administration of antioxidants has been observed to attenuate ALI in several models, including hemorrhagic shock, IIR and sepsis (10–12).

Mast cells are widely distributed in the lungs in order to maintain homeostasis of respiratory function. However, mast cell degranulation can exacerbate ALI, as observed in our previous study, which demonstrated that inhibiting the activation of mast cells alleviated IIR-induced lung injury and reduced the inflammatory response in a rodent model (13). ROS have been observed to mediate mast cell degranulation *in vitro* (14), and excessive activation of mast cells contributes to allergic and inflammatory diseases of the respiratory system (15,16). However, there remains no direct evidence demonstrating the role of mast cell activation by oxidative stress in IIR-induced ALI. The present study hypothesized that mast cell activation exacerbates IIR-mediated ALI primarily through oxidative stress.

Sevoflurane (SEV) is one of the most commonly used inhaled anesthetics (17,18). Preconditioning with SEV has been demonstrated to protect against ischemia-reperfusion injury in various organs, particularly in the lungs and brain (19,20). It has been suggested that the antioxidant and anti-inflammatory properties of SEV contribute to protection against sepsis, ventilation-induced lung injury and lipopolysaccharide-induced lung injury (21–23). However, the role of SEV preconditioning in IIR-mediated ALI remains to be elucidated. The present study investigated whether SEV preconditioning prevents against IIR-induced ALI through inhibition of the synergistic actions between mast cell activation and oxidative stress.

## Materials and methods

### Animals and treatment

**Figure 1:**
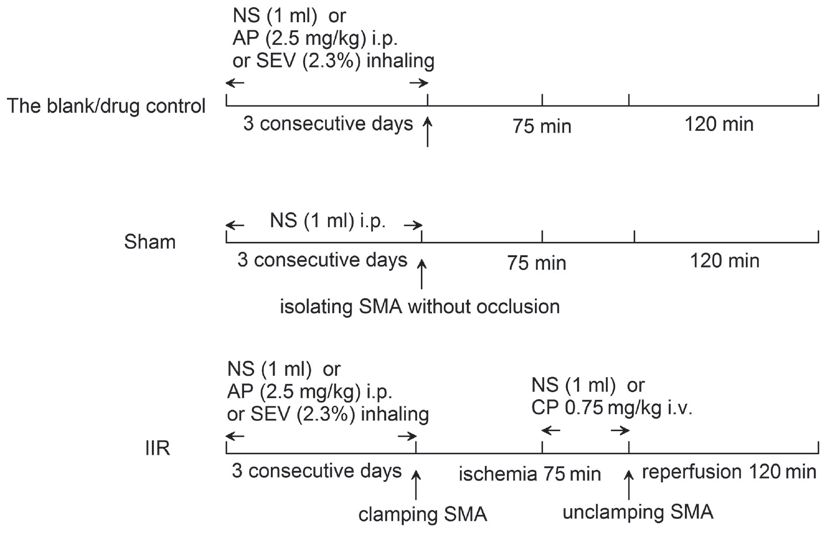
Experimental procedures. The blank/drug control group received no surgery and were administered with NS, SEV or AP only. In the sham group, surgery, involvong isolation of the SMA was performed, without occlusion. The IIR animals received pretreatment with NS, SEV or AP prior to SMA occlusion surgery, followed by treatment with CP or NS prior to 2 h reperfusion. The NS (1 ml) and AP (2.5 mg/kg) were administered via intraperitoneal injection and 2.3% SEV was provided by inhalation for three consecutive days prior to surgery. CP (0.75 mg/kg) was administrated via the caudal vein 5 min prior to reperfusion. NS, normal saline; SEV, sevoflurane; AP, apocynin; SMA, superior mesenteric artery; IIR, intestinal ischemia-reperfusion; CP, compound 48/80.

A total of 60 female adult Sprague-Dawley rats (weighing 200–250 g) were obtained from the Animal Centre of Sun Yat-sen University (Guangzhou, China). The use and care of animals, in addition to the experimental and surgical procedures, were reviewed and approved by the Institutional Animal Care and Use Committee of Sun Yat-Sen University and the Ethics Committee of Sun Yat-Sen University. The animals were housed under a 14 h:10 h light-dark cycle at room temperature between 18 and 26°C and 60–70% humidity. Food and water were available *ad libitum*. The rats were randomly divided into 10 groups (6 rats/group): Normal saline (NS), SEV, apocynin (AP), sham, IIR, IIR + compound 48/80 (CP), SEV + IIR, SEV + IIR + CP, AP + IIR and AP + IIR + CP). The treatments administered to these groups was as follows: NS group, 1 ml NS only; SEV group, 2.3% SEV inhalation only; iii) AP group, 2.5 mg/kg AP only; sham, administration of 1 ml NS each day for 3 days prior to surgical isolation of the superior mesenteric artery (SMA) without occlusion; IIR, administration of 1 ml NS each day for 3 days prior to IIR, established by occluding the SMA for 75 min, followed by 2 h reperfusion; IIR + CP, IIR with administration of CP (0.75 mg/kg) via the caudal vein 5 min prior to reperfusion; vii) SEV + IIR, IIR following exposure to 2.3% SEV each day for 3 days; SEV + IIR + CP, IIR + CP following exposure to 2.3% SEV each day for 3 days; AP + IIR, IIR following pretreatment with AP each day for 3 days; and AP + IIR + CP, IIR + CP following pretreatment with AP each day for 3 days. In the NS, AP and SEV groups, the animals did not undergo surgery, but received saline (1 ml; KeyGen Biotech. Co., Ltd., Nanjing, China) or AP (2.5 mg/kg; Sigma-Aldrich, St. Louis, MO, USA) via intraperitoneal injection, or 2.3% SEV (Maruishi Pharmaceutical Co., Ltd., Osaka, Japan) by inhalation. In the sham group, the SMA was isolated, but not clamped; in the IIR group, all rats received SMA separation and clipping for 75 min, followed by 2 h reperfusion. For the groups treated with NS, AP or SEV, the rats were injected with NS (1 ml) or AP (2.5 mg/kg), or administered with 2.3% SEV via inhalation for three consecutive days prior to surgery. For the groups treated with CP, 0.75 mg/kg CP (Sigma-Aldrich) was injected via the caudal vein 5 min prior to reperfusion. The rats in the remaining IIR groups received the same volume of normal saline (1 ml). The experimental procedure used in the present study is presented in Fig. 1. A heating lamp was used to maintain the body temperatures of the rats. The optimal doses of SEV (2.3%), AP (2.5 mg/kg) and CP (0.75 mg/kg) were adjusted, in accordance with those previously described (24–26) with modifications.

### Preparation of tissue specimens

The rats were sacrificed through overdose of pentobarbital (70 mg/kg; intraperitoneal injection; Sigma-Aldrich) 2 h after reperfusion. Blood samples (2 ml) were obtained from the abdominal aorta and centrifuged (Centrifuge 5804; Eppendorf, Hamburg, Germany) at 1,699 × g for 15 min at 4°C, and the resultant plasma samples were stored at -80°C until analysis. A thoracotomy was immediately performed and the right upper lung was removed, fixed in 10% formaldehyde (Sigma-Aldrich) and embedded in paraffin (Leica Biosystems, Nussloch, Germany) for sectioning. The middle lobe of the right lung was removed and used to measure the wet/dry (w/d) weight ratio. The inferior lobes of the right and left lungs were removed and preserved in liquid nitrogen (Guangzhou Pearl River Industrial Gases Co. Ltd., Guangzhou, China) for further analysis of oxidative stress, mast cell activation and inflammatory indicators.

### Measurement of lung w/d ratio

The middle lobes of the right lungs were weighed (weight_wet_) immediately using a precision balance [Mettler-Toledo (Schweiz) GmbH, Greifensee, Switzerland], and re-weighed (weight_dry_) following incubation at 95°C in an oven (876–1 Vacuum Drying Oven; Nantong Science Instrument Factory, Nantong, China) for 24 h. The w/d ratio was calculated as follows: w/d = weight_wet_ / weight_dry_.

### Histopathological examination

The lung tissues embedded in paraffin were dissected into 4-*μ*m microsections (ZQP-86; Zhejiang Xiangshan Scientific Precision Instrument Factory, Xiangshan, China), which were stained and observed under a light microscope (Eclipse E200; Nikon, Tokyo, Japan). Hematoxylin and eosin (Beyotime Institute Biotechnology, Shanghai, China) staining was used to assess pathological injury, while staining with toluidine blue (Beijing Leagene Biotech Co., Ltd., Beijing, China) was used to count the number of mast cells. The degree of lung injury was assessed using a scoring system, described by Hofbauer *et al* (27). According to this scoring system, edema of the alveoli and mesenchyme, intra-alveolar inflammatory cell infiltrates, alveolar hemorrhage and atelectasis were graded on a scale between 0 and 4. The grades were as follows: 0, normal, <15% of space occupied by tissue and >85% occupied by alveolar space; 1, 15%–25% of space occupied by tissue and 75%-85% occupied by alveolar space; 2, 25%–50% occupied by tissue and 50%–75% occupied by alveolar space; 3, 50%–75% occupied by tissue and 25%–50% occupied by alveolar space; and 4, 75%–100% occupied by tissue and 0%–25% occupied by alveolar space. For the sections stained with toluidine blue, cells containing blue-purple granules in the cytoplasm were considered to be mast cells.

### Detection of the levels of β -hexosa minidase

β-hexosaminidase is one of the specific enzymes synthesized by mast cells, and mast cell activation is accompanied by the release of histamine and β-hexosaminidase. While histamine is metabolized rapidly, β-hexosaminidase is metabolized less readily and can be used as an index of mast cell activation (28). In the present study, the lung tissues were homogenized with cold normal saline and the homogenates were centrifuged at 1,699 × g for 15 min at 4°C. The supernatants were then transferred into fresh tubes for detection. The activities of β-hexosaminidse in the lung tissue homogenates and blood samples were detected using a β-hexosaminidase kit, according to the manufacturer’s instructions (Nanjing KeyGen Biotech. Co., Ltd.).

### Detection of the levels of malondialdehyde (MDA) and hydrogen peroxide (H_2_O_2_)

The levels of MDA and H_2_O_2_ in the homogenates of the lung tissues were measured using the MDA Detection kit and the H_2_O_2_ Detection kit, according to the manufacturer’s instructions (KeyGen Biotech. Co., Ltd.).

### Detection of the activity levels of interleukin (IL-6) and myeloperoxidase (MPO) activity

The concentration of IL-6 is an independent marker for the inflammatory response. MPO is an enzyme induced by activated neutrophils and is considered an indicator of neutrophil infiltration (29,30). The activities of IL-6 and MPO in the lung tissues were measured using the IL-6 Detection kit and the MPO Detection kit (KeyGen Biotech Co., Ltd.), according to the manufacturer’s instructions.

### Western blotting

Prosurfactant protein C (proSP-C) is the precursor of surfactant protein C, which is exclusively produced in alveolar type II cells to prevent lung collapse (31). Trypatse is one of the characteristic markers of mast cell activation (32). The expression of NADPH oxidase reflects the level of oxidative stress, and p47^phox^ and gp91^phox^ are subunits of NADPH oxidase (33). Intercellular adhesion molecule-1 (ICAM-1, CD54) is an important early marker of immune activation and response (34). Western blot analyses were performed, as previously described (24). The primary antibodies used in the present study were as follows: Anti-ICAM-1 mouse monoclonal antibody (1:500; sc-8439; Santa Cruz Biotechnology, Inc., Santa Cruz, CA, USA), antitryptase rabbit polyclonal antibody (1:500; sc-32889; Santa Cruz Biotechnology, Inc.), anti-proSP-C polyclonal rabbit antibody (1:1000; AB3786; EMD Millipore, Billerica, MA, USA), anti-p47^phox^ polyclonal rabbit antibody (1:1,000; sc-14015; Santa Cruz Biotechnology, Inc.) and anti-gp91^phox^ polyclonal rabbit antibody (1:1,000; sc-20782; Santa Cruz Biotechnology, Inc.). The images were analyzed used ImageJ software, version 1.41 (National Institutes of Health, Bethesda, MA, USA).

### Statistical analysis

All data are expressed as the mean ± standard deviation. Statistical analyses were performed using SPSS software, version 13.0 (SPSS Inc., Chicago, IL, USA). The statistical significance of differences between the groups were evaluated by one-way analysis of variance followed by Bonferroni’s post-hoc test for unpaired values. P<0.05 was considered to indicate a statistically significant difference.

## Results

### SEV and AP increase antioxidant capacity and inactivate mast cells in normal rat lungs

**Figure 2:**
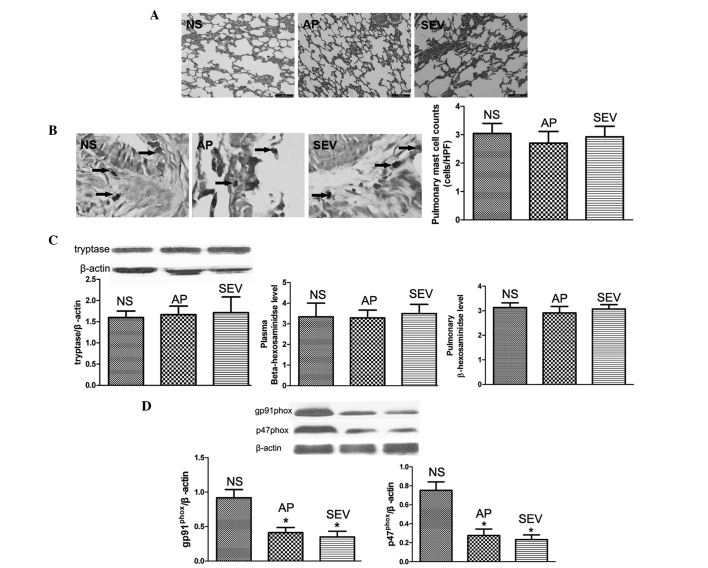
SEV and AP increases antioxidant capacity and inactivates mast cells in normal rat lungs. (A) Histological images of lung morphology. The lung sections were stained with hematoxylin and eosin and visualized at magnification, ×200. (B) Mast cell counts in the lung tissues. The lung sections were stained with toluidine blue. The blue-purple granules in the cytoplasm indicated positive staining for mast cells (arrows). (C) Expression levels of tryptase in the lung tissues and levels of β-hexosaminidase in the plasma and lung tissues. (D) Expression levels of p47^phox^ and gp91^phox^ in the lung tissues were determined using western blot analysis (n=3). All values are expressed as the mean ± standard deviation, n=6 per group. ^*^P<0.05, vs. NS group. SEV, sevoflurane; AP, apocynin; NS, normal saline.

As shown in Fig. 2A, the pulmonary structures were normal following treatments with NS, SEV and AP for 3 days. Treatment with SEV or AP did not significantly change the number of mast cells (Fig. 2B), expression of tryptase or the levels of β-hexosaminidase in either the plasma or the lung tissues (Fig. 2C). However, the expression levels of p47^phox^ and gp91^phox^ were downregulated in the lungs (P<0.05), inhibiting NAPDH enzyme activity (Fig. 2D).

### SEV and AP attenuate IIR-induced lung injury

**Figure 3:**
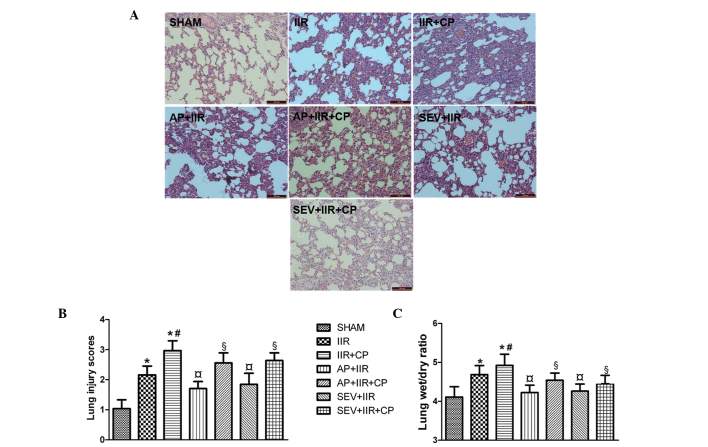
SEV and AP pretreatment attenuates IIR-induced lung injury. (A) Histological images of lung morphology. The lung sections were stained with hematoxylin and eosin and visualized at magnification, ×200. (B) Lung histopathology evaluation scores. (C) Wet/dry weight ratio of the right middle lobe of the lung. All values are expressed as the mean ± standard deviation, n=6 per group. ^*^P<0.05, vs. sham group; ^#^P<0.05 vs. IIR group; ^§^P<0.05 vs. IIR + CP group. SEV, sevoflurane; AP, apocynin; IIR, intestinal ischemia-reperfusion; CP, compound 48/80.

As shown in Fig. 3A, the lung structures in the sham group were normal. IIR resulted in severe damage to the lungs, with collapse of the alveoli, interstitial edema, haemorrhage in the alveoli and mesenchyme, neutrophil infiltration and atelectasis. In addition, treatment with CP aggravated IIR-induced lung injury. Pretreatment with SEV and AP significantly prevented the lung damage induced by IIR and IIR + CP. The pathological injury score and w/d ratio in the lungs were in accordance with the pathological changes observed under the light microscope (Fig. 3B and C).

### SEV and AP decrease the downregulation in the expression of proSP-C induced by IIR and IIR + CP

**Figure 4:**
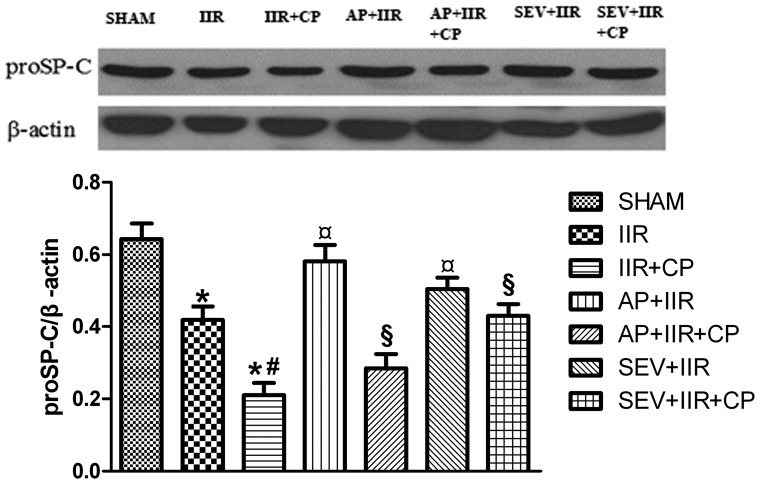
SEV and AP protect type II alveolar epithelial cells from IIR-induced injury. Expression levels of ProSP-C and semi-quantitation of the protein levels of proSP-C protein density by western blot analysis using ImageJ software (n=3). All values are expressed as the mean ± standard deviation. ^*^P<0.05, vs. sham group; ^#^P<0.05, vs. IIR group; ^§^P<0.05, vs. IIR + CP group. SEV, sevoflurane; AP, apocynin; IIR, intestinal ischemia-reperfusion; proSP-C, prosurfactant protein C; CP, compound 48/80.

The expression levels of proSP-C were significantly reduced in the IIR group and were reduced further in the IIR + CP group compared with the sham group (Fig. 4). SEV and AP inhibited the downregulated expression of proSP-C induced by IIR and IIR + CP. These results suggested that IIR caused damage to type II alveolar epithelial cells, that mast cell degranulation exacerbated the damage, and that SEV and AP protected the type II alveolar epithelial cells from the injury induced by IIR and mast cell degranulation.

### SEV and AP inhibit the IIR-induced activation of mast cells

**Figure 5:**
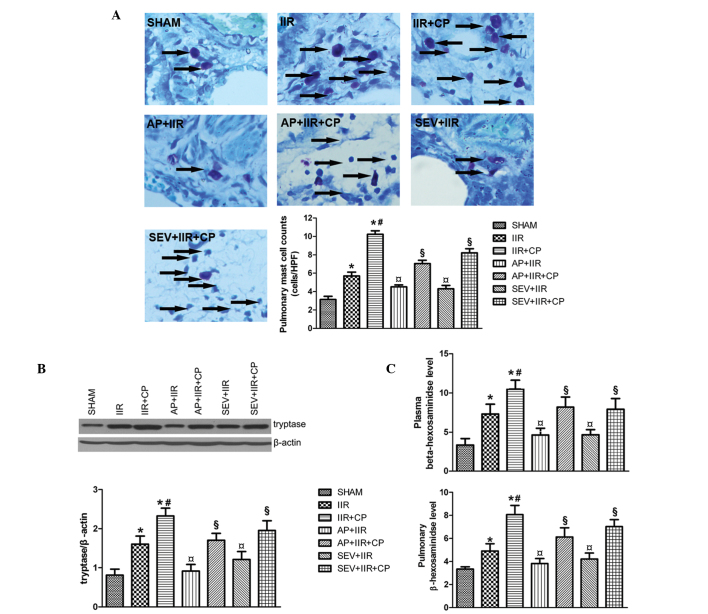
SEV and AP inhibit the IIR-induced activation of mast cells. (A) Mast cell counts in the lung tissues. The lung sections were stained with toluidine blue. The blue-purple granules in the cytoplasm indicate positive staining for mast cells (arrows). (B) Expression levels of tryptase and semi-quantitation of the protein levels of tryptase from western blot analysis using ImageJ software (n=3). (C) Levels of β-hexosaminidase level in the plasma and lung tissues. All values are expressed as the mean ± standard deviation, n=6 per group. ^*^P<0.05, vs. sham group; ^#^P<0.05, vs. IIR group; ^§^P<0.05 vs. IIR + CP group. SEV, sevoflurane; AP, apocynin; IIR, intestinal ischemia-reperfusion; CP, compound 48/80; HPF, high power field.

As shown in Fig. 5, IIR resulted in an increase in the number of mast cells and expression levels of tryptase in the lung tissues, and the levels of β-hexosaminidase in the lungs and plasma. CP aggravated these changes. Pretreatment with SEV and AP effectively alleviated the changes induced by IIR and IIR + CP.

### SEV and AP attenuate IIR-induced oxidative stress

**Figure 6:**
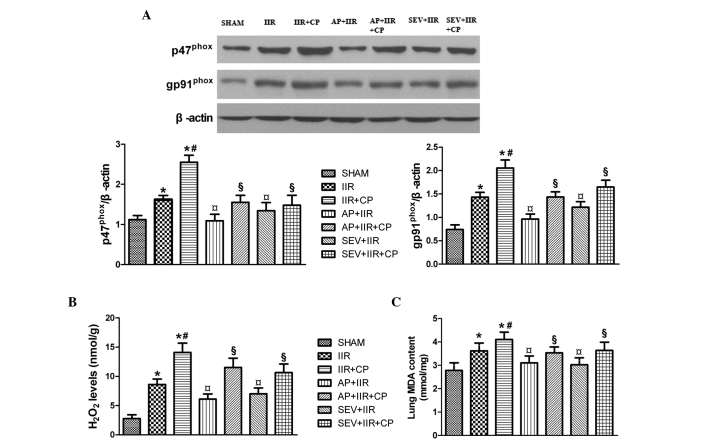
SEV and AP attenuate IIR-induced oxidative stress. (A) Expression levels of p47^phox^ and gp91^phox^ and semi-quantitation of the protein levels of tryptase from western blot using ImageJ software (n=3). (B) Levels of H_2_O_2_ in the lung tissues. (C) Levels of MDA in the lung tissues. All values are expressed as the mean ± standard deviation, n=6 per group. ^*^P<0.05, vs. sham group; ^#^P<0.05, vs. IIR group; ^§^P<0.05 vs. IIR + CP group. SEV, sevoflurane; AP, apocynin; IIR, intestinal ischemia-reperfusion; H_2_O_2_, hydrogen peroxide; MDA, malondialdehyde; CP, compound 48/80.

NADPH oxidase is crucial for ROS generation, and p47^phox^ and gp91^phox^ are the predominant subunits of NADPH oxidase (35). As shown in Fig. 6, IIR increased the expression levels of p47^phox^ and gp91^phox^ in the lungs, which were further upregulated by CP. Pretreatment with SEV and AP significantly reversed this upregulation in the expression levels of p47^phox^ and gp91^phox^. The changes observed in the levels of H_2_O_2_ and MDA were consistent with the changes observed in the expression of p47^phox^ and gp91^phox^. These results suggested that SEV and AP alleviated the oxidative injury in the lungs, induced by IIR, by inhibiting the activity of NADPH oxidase.

### SEV and AP inhibit the IIR-induced inflammatory response

**Figure 7:**
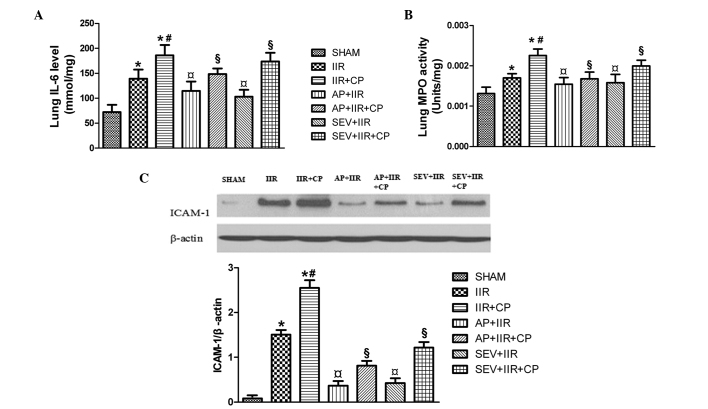
SEV and AP inhibit the IIR-induced inflammatory response. (A) Levels of IL-6 in the lung tissues. (B) Activity of MPO in the lung tisues. (C) Expression levels of ICAM-1 and semi-quantitation of the protein levels of ICAM-1 from western blot analysis using ImageJ software (n=3). All values are expressed as the mean ± standard deviation, n=6 per group. ^*^P<0.05, vs. sham group; ^#¤^P<0.05 vs. IIR group; ^§^P<0.05 vs. IIR + CP group. SEV, sevoflurane; AP, apocynin; IIR, intestinal ischemia-reperfusion; IL-6, interleukin-6; MPO, myeloperoxidase; ICAM-1, intercellular adhesion molecule-1; CP, compound 48/80.

As shown in Fig. 7, IIR increased the levels of IL-6, activity of MPO and the protein expression levels of ICAM-1 levels, and these were increased further by CP. Pretreatment with SEV and AP effectively reduced the levels of IL-6, activity of MPO and protein expression of ICAM-1, which was induced by IIR and IIR + CP. These results suggested that SEV and AP inhibited IIR-induced lung injury by inhibiting inflammatory responses.

## Discussion

The present study demonstrated that oxidative stress is important in IIR-induced ALI, evidenced as significant elevations in the expression levles of p47^phox^ and gp91^phox^ in the lungs, in addition to increases in the levels of H_2_O_2_ and MDA. Furthermore, mast cells were found to exacerbate IIR-mediated ALI, revealed by significant increases in the pathological injury score and w/d weight ratio of the lungs and reductions in the expression of proSP-C in the lungs. Notably, pretreatment with the antioxidant, AP, or with SEV not only attenuated ALI, but also inhibited mast cell degranulation-mediated exacerbation in the presence of CP. To the best of our knowledge, the present study was the first to demonstrate the ability of SEV to limit ALI by inhibiting the synergistic action between oxidative stress and mast cell activation. The results offer promising therapeutic benefits against IIR-mediated ALI.

Several previous studies have reported that oxidative stress and uncontrolled inflammation contribute to the process of IIR-mediated ALI (36–38). In the present study, significantly increased expression levels of the p47^phox^ and gp91^phox^ NADPH enzymes and increased levels of MDA and H_2_O_2_ were observed in the IIR group. In addition, pretreatment with the NADPH oxidase inhibitor, AP, attenuated ALI by significantly reducing the protein expression levels of p47^phox^ and gp91^phox^ and the levels of MDA and H_2_O_2_, further suggesting that oxidative stress is central in the process of ALI.

Several previous studies have demonstrated that mast cells are important in the process of ALI in different models, including sepsis, hemorrhagic shock and small IIR injury (39–41). Mast cells, which contain large quantities of cytokines and proteases, are widely distributed around the capillaries and lymph vessels of the connective tissue in the respiratory system. When activated, the released mediators are able to exacerbate IIR-induced ALI. Several factors contribute to mast cell degranulation (26,42,43). A previous study demonstrated that phenyl N-tertbutylnitrone, a ROS scavenger, reduced the enhancement of peritoneal mast cell activity induced by supernatant from colonic biopsies, indicating that ROS is involved in mast cell activation *in vitro* (44). However, the precise role of oxidative stress in mast cell degranulation during the process of IIR-mediated ALI remains to be fully elucidated. In the present study, AP, an NADPH oxidase inhibitor, attenuated IIR-induced ALI and oxidative stress compared with the IIR group. AP also inhibited this exacerbation in the presence of the mast cell activator, CP. These observations suggested that mast cell activation, induced by oxidative stress, is pivotal in IIR-mediated ALI.

SEV is one of the most commonly used volatile anesthetics and, in addition to its anesthetic effects, several studies have demonstrated that SEV exhibits antioxidant and anti-inflammatory properties (20,21). Preconditioning with SEV has been demonstrated to protect the heart, kidneys and lungs against ischemia-reperfusion injury *in vitro* and *in vivo* (45–49). In agreement with previous studies, the results of the present study indicated that SEV preconditioning attenuated IIR-mediated ALI by downregulating oxidative stress and the inflammatory response, as did treatment with AP. These results further confirmed SEV preconditioning as beneficial against ischemia-reperfusion injury.

Uncontrolled inflammation also contributes to the process of IIR-mediated ALI (50,51). In line with the previous studies, the observations of the present study also demonstrated that the activity of MPO, and the expression levels of ICAM-1 and IL-6 were significantly increased following IIR challenge, and these elevations were further enhanced in the presence of CP. The findings indicated that oxidative stress and mast cell activation, in addition to their synergistic action, contributed to the pulmonary inflammatory response and were important in the process of IIR-induced ALI. Similarly, preconditioning with SEV and AP inhibited the exacerbations induced by IIR combined with CP, therfotr, the results suggested that SEV protected against IIR-mediated ALI by inhibiting the synergistic effects of mast cells and oxidative stress (52–55).

In conclusion, SEV was observed to attenuate IIR-induced lung injury by inhibiting mast cell activation, minimizing oxidative damage and suppressing their synergistic effects. These results may have an implication in the clinical treatment of IIR-mediated ALI.
